# Parametric Model of Combination Therapy for Non-Hodgkin Lymphoma

**DOI:** 10.1371/journal.pone.0051736

**Published:** 2012-12-14

**Authors:** Robert F. Weiss, Merlin G. Miller, John F. Cronin, Harvey H. Hensley, Indira D. Joshi, Mitchell R. Smith

**Affiliations:** 1 Back Bay Biosciences, Boston, Massachusetts, United States of America; 2 Computer Services, Physical Sciences Inc., Andover, Massachusetts, United States of America; 3 Cancer Biology, Fox Chase Cancer Center, Philadelphia, Pennsylvania, United States of America; 4 Medical Oncology, Fox Chase Cancer Center, Philadelphia, Pennsylvania, United States of America; University of Navarra, Center for Applied Medical Research, Spain

## Abstract

The development and clinical testing of drug combinations for the treatment of Non-Hodgkin Lymphoma (NHL) and other cancers has recently shown great promise. However, determining the optimum combination and its associated dosages for maximum efficacy and minimum side effects is still a challenge. This paper describes a parametric analysis of the dynamics of malignant B-cells and the effects of an anti-sense oligonucleotide targeted to BCL-2 (as-bcl-2), anti-CD-20 (rituximab) and their combination, for a SCID mouse human lymphoma xenograft model of NHL. Our parametric model is straightforward. Several mechanisms of malignant B-cell birth and death in the nodal micro-environment are simulated. Cell death is accelerated by hypoxia and starvation induced by tumor scale, by modification of anti-apoptosis with as-bcl-2, and by direct kill effects of rituximab (cell kill by cytotoxic immune cells is not included, due to the absence of an immune system in the corresponding experiments). We show that the cell population dynamics in the control animals are primarily determined by K*, the ratio of rate constants for malignant cell death, K**_d_**, and cell birth, K**_b_**. Tumor growth with independent treatments is reproduced by the model, and is used to ***predict*** their effect when administered in combination. Malignant cell lifetimes are derived to provide a quantitative comparison of the efficacy of these treatments. Future experimental and clinical applications of the model are discussed.

## Introduction

The development and clinical testing of drug combinations for the treatment of non-Hodgkin Lymphoma (NHL) and other cancers has recently shown great promise [1]. However, determining the optimum combination and its associated dosages for maximum efficacy and minimum side effects is still a challenge. This study addresses several questions:

1. Can a parametric model quantitatively simulate the separate effects of as-bcl-2 and anti-CD-20 compared to the control?
2. Can the benefits of each therapy relative to the control be quantitatively measured in terms of reduced malignant cell lifetimes?
3. Can the model quantitatively simulate the ***combined*** effects of these therapies without introduction of additional parameters?
4. Can the model use the independently determined key parameters for individual therapies to ***predict*** their combined efficacy?
5. Can the quantitative results suggest the relative importance of the separate mechanisms simulated in the model?

Affirmative answers to these questions will validate the model and provide a tool for the design of dedicated animal experiments to identify optimum combinations of drugs. They may also assist with the planning of future clinical trials in humans using similar drug combinations.

Data from experiments in which human lymphoma cells are grown in immuno-deficient SCID mice that are then treated with as-bcl-2 and monoclonal antibody suggest that combination therapy has a qualitatively larger effect on malignant cell populations than either treatment alone [2]. However, it is not clear if the observed combined efficacy is synergistic, or predictable. If the individual treatments are synergistic, a parametric model that includes their individual biological mechanisms should be able to simulate their combined efficacy.

In the next section, we describe the experimental procedure and data reduction process, in which the tumor volumes are carefully measured by summing planar MRI images. The following section describes a parametric model that explicitly connects each independent therapy to one or more terms in the model. We then apply the full parametric equation to predict the efficacy of combined treatment and compare these predictions to the combined therapy data in the following section. Agreement between the model and data will provide an initial validation of the model and a quantitative evaluation of combination treatment.

In the final section, the model is used to derive average cell lifetimes from the mouse tumor volume data as a metric for the effectiveness of each therapy. We then discuss these results, provide tentative answers to the questions posed above, and suggest future directions and applications.

## Materials and Methods

### Experimental Methods

We examined the effects of combination therapies on the DoHH2 human lymphoma cell line (0.25×10^6^ + 2.5 mg Matrigel/0.4 ml PBS) injected subcutaneously into immune deficient mice. DoHH2, a t(14;18)+ transformed lymphoma cell line, was obtained from the Deutsche Sammlung von Mikroorganismen und Zellkulturen GmbH (DMSZ, German Collection of Microorganisms and Cell Cultures, Braunschweig, Germany).

These cells were allowed to grow until a time, typically ten days, when mass was palpable. Measurement of tumor volume was performed by multiple “slice” MRI whose minimum resolution, limited by voxel size, was approximately 0.2 mm. The outline of the tumor was clearly visible, and the cross-sectional area of a slice of the tumor was readily calculated. A set of such slices was then summed to determine total tumor volume at each designated day of the experiment. The first measurements were made an additional five days after the animals were variably injected intraperitoneally as before [2] with:

**mut-bcl-2**: an oligonucleotide with no activity, administered alone (the “control”) or in combination with anti-CD-20 as a single dose of 200 micrograms per gram.
**anti-sense bcl-2**: an antisense oligonucleotide to down-regulate ***bcl-2*** that should enhance malignant cell apoptosis without affecting cell birth rates, thereby causing malignant cells to be “sensitized” and become more vulnerable to kill mechanisms, administered as a single dose of 200 micrograms per gram.
**anti-CD-20**: a monoclonal antibody (***rituximab***), should preferentially, and directly, kill malignant cells (note that indirect kill by cytotoxic T-cells was not likely in these immune deficient mice, although some residual NK cells may have been present), administered as a single dose of 5 micrograms per gram (higher doses have been administered in other experiments not reported here).
**anti-sense bcl-2** and **anti-CD-20** combination: single doses administered as above.

Ref [2] justifies the dose schedule of as- bcl-2 and control oligos, as well as that for anti-CD20, and demonstrates effects on both DoHH2 and FSCCL cell lines. Further, Ref. [3] confirms in vivo down-regulation of bcl-2 by anti-sense oligos. In vitro cell line data was reported in Ref. [4].

**Figure 1:**
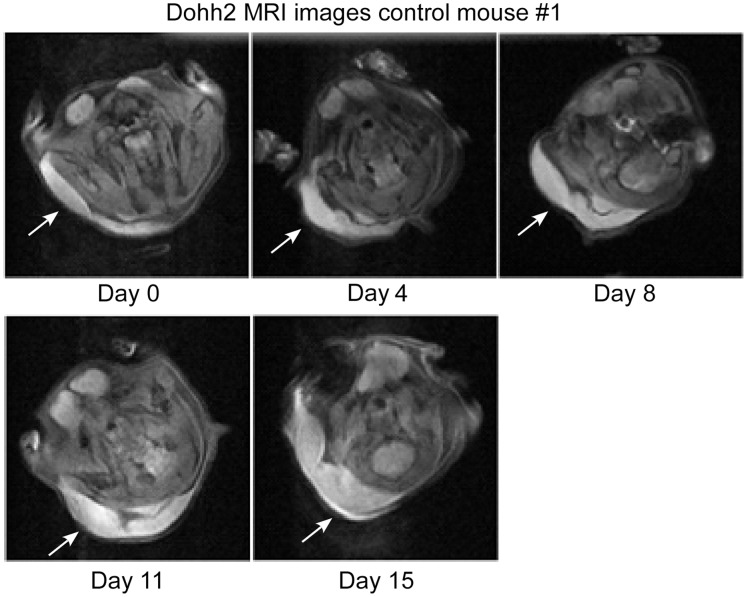
Maximum diameter tumor slice (bright area) for a control mouse.

**Figure 2:**
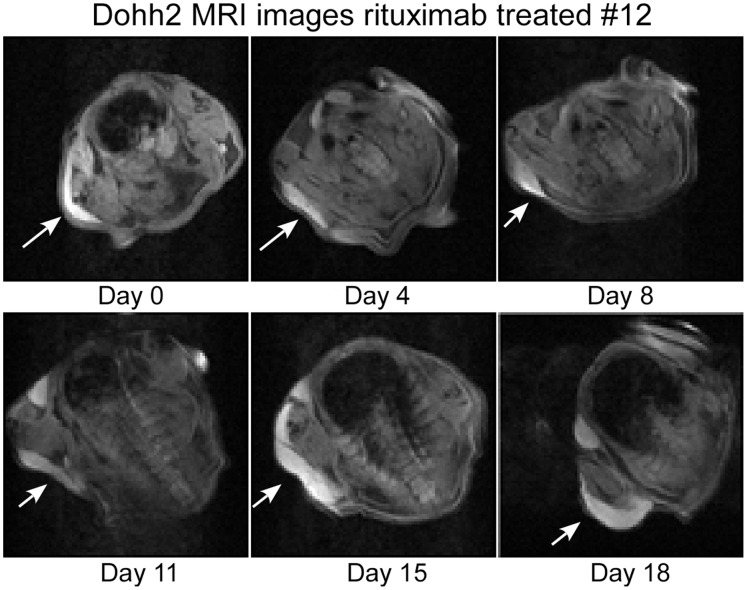
Maximum diameter tumor slice for a rituximab treated mouse.

**Figure 3:**
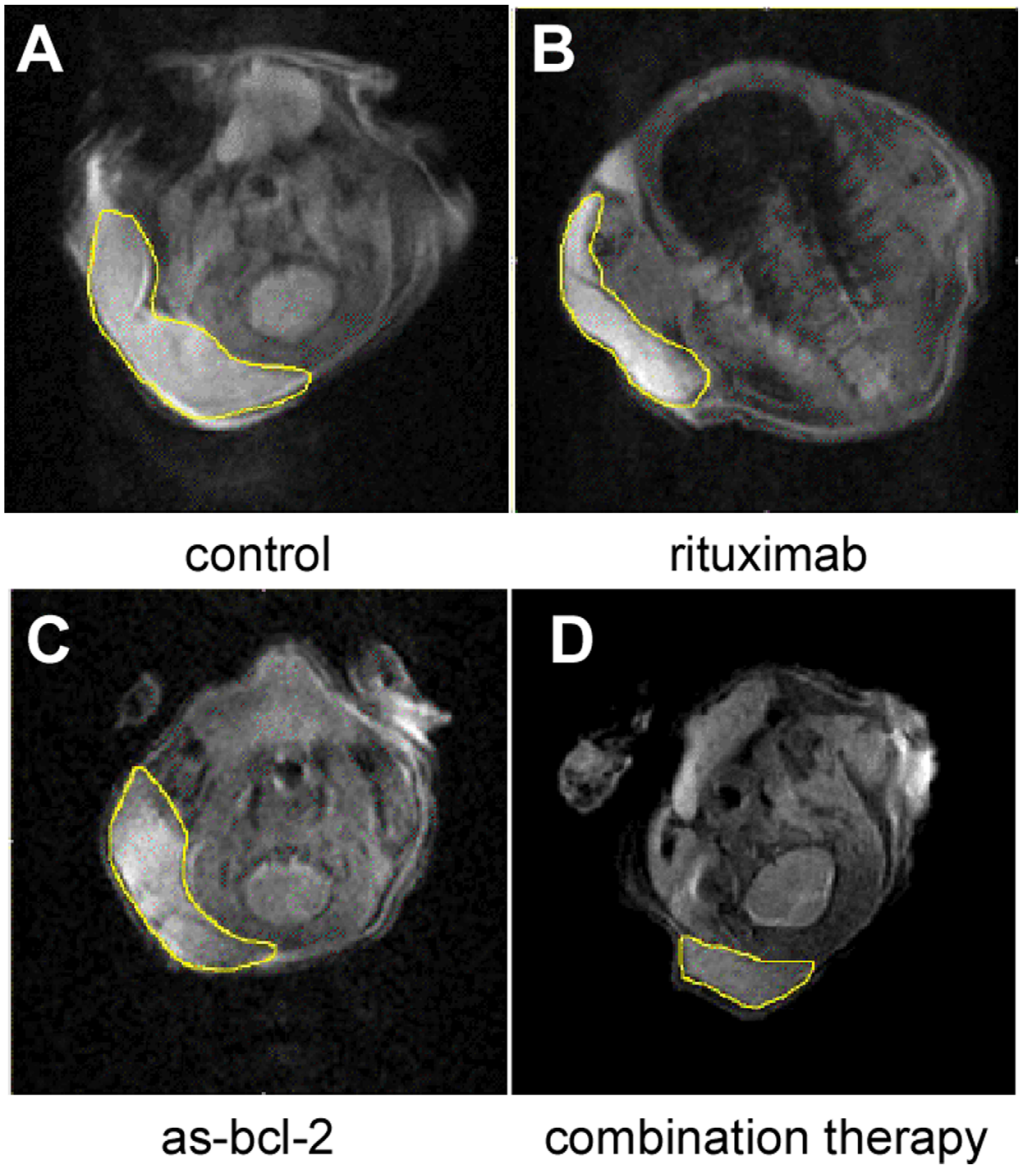
MRI "slices" on day 15 for animals in the a) control, b) rituximab, c) as-bcl-2 and d) combination therapy groups.

A set of five mice comprised each group and tumor volumes were followed for up to 23 days, yielding between four and five data points per experiment. Examples of MRI “slices” at maximum tumor girth are shown for a control animal and a mouse treated with rituximab alone in Figure 1 and Figure 2, respectively. These images are axial views through the chest of the mouse. The large signal void is the mouse’s lungs. The tumor is the bright area on the left hand side of the image. In Figure 3, we compare sample images taken on day 15 for animals in the control, rituximab, as-bcl-2 and combination therapy groups. The tumors are outlined in yellow for improved visibility.

Tumor volumes were measured by outlining the tumor manually using imaging system software, and adding the volumes outlined on each slice. Each slice is 0.5 mm thick, and has a 2.56 cm field of view with a 128×128 image matrix, giving a 0.2 mm in-plane resolution. A T2 weighted spin echo pulse sequence was used. The tumor xenografts were quite bright with these parameters. As can be seen from Figure 1 and Figure 2, the tumors tended to grow in a flat layer beneath the skin, and did not protrude from the animal sufficiently for caliper measurements to be accurate. With MRI protocols requiring only a few minutes to complete for each animal, such methods (where available) would likely be feasible even for conventional subcutaneous models, providing more accurate tumor volume measurements while requiring a greater, but not prohibitive, increase in effort.

All work was conducted under approval from our Institutional Animal Care and Use Committee (IACUC). No tumors grew in size to exceed 10% of bodyweight. Animals were monitored daily for health and activity, including any signs of bleeding or ulceration of the tumor sites (which were not observed in our model).

**Figure 4:**
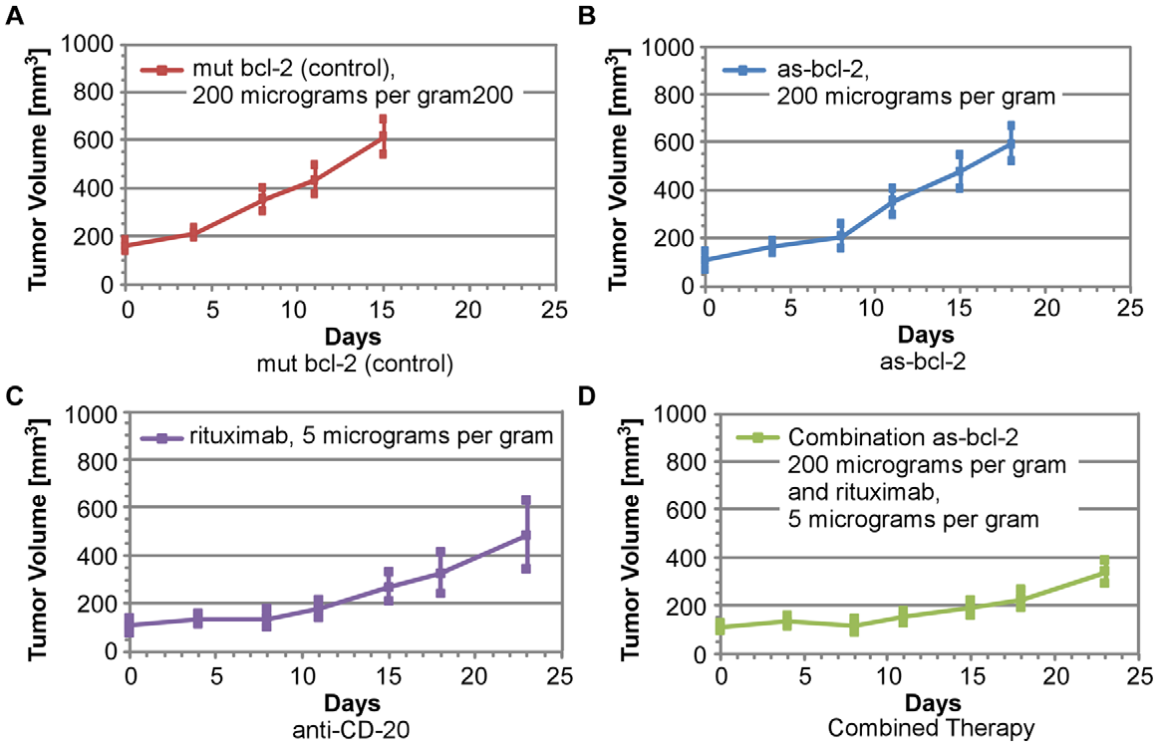
Average tumor volume measurements for each data set.

**Figure 5:**
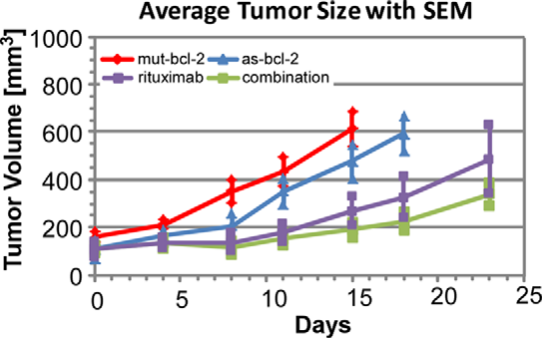
Comparison of average tumor growth histories with SEMs indicated.

Average tumor volumes and the related Standard Error of the Mean (SEM) for all twenty experiments are plotted in Figures 4a, b, c and d. Analysis of individual mouse responses would be of interest, but the current data base is insufficient to support such analysis. We note that average initial tumor sizes were 158, 114, 125 and 109 mm^3^ for the mut-bcl-2 (control), as-bcl-2, rituximab and combination as-bcl-2/rituximab groups, respectively. In the modeling described in the next section, we will non-dimensionalize all tumor cell populations by their initial values, which are assumed to be proportional to measured tumor volumes divided by *their* initial values (which are not statistically different, as the *initial* SEMs overlap) to compare their subsequent growth and response to therapy. While we recognize the limitations inherent in small experimental samples, the slight overlap of the SEMs in Figure 5 suggests that the average tumor volumes are both statistically separable and responsive to as-bcl-2, anti-CD-20 and combined therapies (experiments at other dosages were statistically analyzed in Ref. [2] and will not be reported here). Furthermore, we believe that the early time data suggests a synergistic effect between as-bcl-2 and anti-CD-20 when they are used in combination. There also appears to be a change in the rate of tumor growth at seven days. The magnitudes and possible causes of this change in net tumor growth rate will be addressed later.

### Parametric Modeling

The rate of change of the malignant tumor cell population with respect to time (t) is equal to the sum of cellular mechanisms that characterize the birth and death rates of population members, the direct kill of population members by various external mechanisms, and other effects that limit the growth or accelerate the death of the population, such as competition for limited resources. The resulting “logistics” - like equation has been successfully applied for decades to the simulation of population dynamics for a broad range of problems [5].

In this model, the birth term is the product of the tumor cell birth rate K**_b_** and its current population, N(t). The death term at low concentrations of malignant cells is the product of tumor cell death rate, K**_d_**, and its current population. The cell death rate is assumed to be ***enhanced*** by a factor f = 1+N(t)/N(0). The N(t)/N(0) term represents the first order influence of hypoxia and starvation, which limit population growth. This factor is related to the “carrying capacity” term used in population growth models of tumors and ecological systems [5].

In the absence of an immune system, indirect kill by cytotoxic T-cells due to rituximab is absent. However, rituximab treatment can also induce direct kill, which we model with a term that is the product of the kill rate, K_K_, a function g(c), where c is the concentration of rituximab relative to body mass, and the current malignant cell population.

Summing these terms (with appropriate sign) yields the basic model:
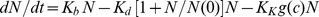
(1)


We non-dimensionalize the malignant cell population, N, by its initial value, N(0), so that the non-dimensional population number is N* = N/N(0). The natural parameter with which to non-dimensionalize elapsed time is K**_b_^−1^**, the inverse of the cell birth rate and the characteristic “e-folding” time, T, for initial exponential growth. Non-dimensional time is then t* = t K**_b_** = t/T. We shall adopt the convention of using K* as the ratio of the inherent malignant cell death rate to cell birth rate, most closely representative of the early stage disease in untreated animals. Thus,
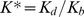



We expect that as-bcl-2 will modify the death rate, and note this by changing K* to **K′ when as-bcl-2 is administered**.

We also define
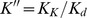
and non-dimensionalize g(c) so that g = 1 when c = 5 micrograms/per gram, the concentration of rituximab in the subject experiments.

Inserting these definitions into Eq (1) yields the non-dimensional model equation:
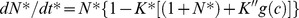
(2)where K* is replaced by K′ when modeling the as-bcl-2 experiments.

The data from which we will derive K″g(c) was taken at a dosage of 5 micrograms per gram. Prediction of the effect of higher dosages will require a functional form for g(c), keeping K″ a constant. This will be the subject of future comparisons with other experimental data taken at Fox Chase Cancer Center.

Our model computation strategy is straightforward: We first determine K*, K′ and K″ from the individual experimental data for mut bcl-2, as-bcl-2 and anti-CD-20. We then predict the combination therapy data with these parameters fixed. This four step calculation, employs Eq. (2) at each step:

1. For the control group (mut-bcl-2), we set K″ = 0, N*(0) = 1, and determine the values of T and K* that best match the data by visual inspection. After many independent trials, and recognizing the limited data base, we decided that a formal “least-squares” or similar fitting process would provide no real improvement to these estimates.
2. For the as-bcl-2 group, we again set K″ = 0 and N*(0) = 1, designate K* as K′, and find K′ using the same approach as above, with T held at the value found in step 1.
3. For the rituximab group, we use K* from step 1, N*(0) = 1, and find K″g(c) with the same approach as above, again holding T at the value found in step 1.
4. For the combination group, we use T from step 1, K′ from step 2, K″ from step 3, N*(0) = 1, and ***predict*** the experimental tumor volume history for this group.

## Results

**Figure 6:**
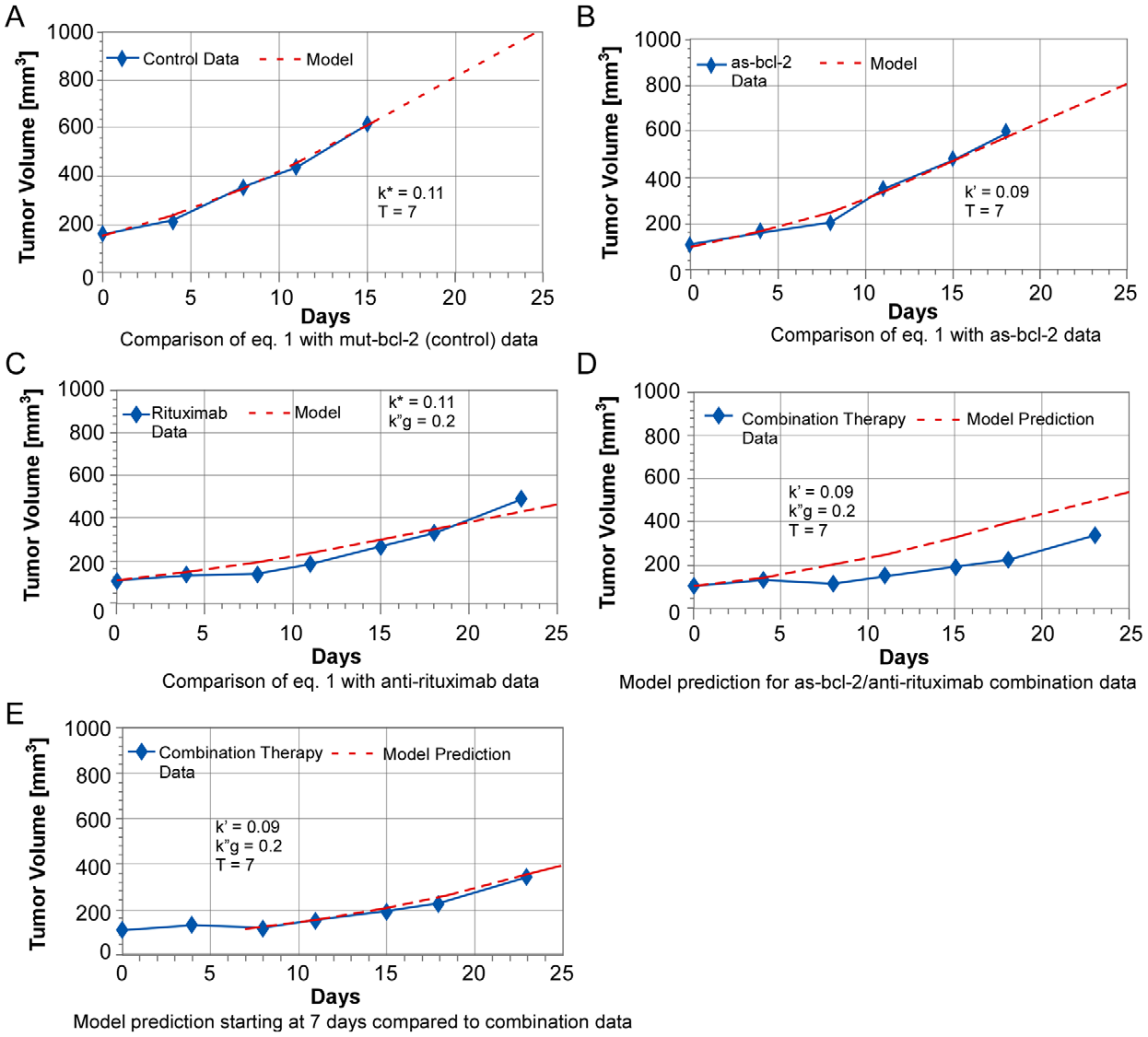
Comparison of parametric model with independent and combination therapies.

As illustrated in Figures 6a, b and c, we obtain reasonable matches between the parametric model and the average tumor volume data for the control experiments and the two independent treatment experiments. The control data fit yields T = 7 days as the characteristic e-folding time for malignant cell growth and K* = 0.11. This value of K* means that the isolated malignant cell death rate is 11% of its birth rate, so that the characteristic lifetime of such cells, L = T/K* = 1/K**_d_**, is approximately 63 days. The parametric model accurately simulates the average mut-bcl-2 (control) data for the entire length of these experiments, and is in reasonable overall agreement with the average as-bcl-2 and anti- CD-20 data as well. However, as shown in Figure 6d, the model does not accurately predict the combination treatment data. This is because the value of K′ (0.09) for the as-bcl-2 data is slightly less than that of K* (0.11) derived for the control, which is the opposite of the anticipated effect. These values are the best fits to the data over the entire experiment, but a value of K′ = 0.15 is required to match the early time data in Figure 4b, c and d, where little growth is observed.

When we initiate the previous model prediction (K* = .11 and K′ = .09) for the combination treatment at 7 days, excellent agreement with the combination therapy data is obtained, as shown in Figure 6e. There are at least two potential explanations for this behavior: 1) anti-CD-20 and combination therapy are most effective at early times, when both as-bcl-2 and anti-CD-20 are in maximum abundance, or 2) the therapy is more effective with the most vulnerable of a heterogeneous population of tumor cells, which are eliminated in the first week, leaving the less vulnerable cells to dominate tumor growth after 7 days. We note that T, the theoretical recovery time for these tumors, is also 7 days.

This analysis of the experimental data suggests that the primary effect of as-bcl-2 occurs during the first week after injection, a period during which little or no tumor growth is observed. The primary anti-tumor agent during this period is clearly anti-CD-20, but its efficacy appears to be enhanced by the simultaneous introduction of as-bcl-2. We will derive a quantitative measure of these “early time” effects in the next section.

## Discussion

We can use the experimentally-deduced parameters to derive a quantitative measure of the early time effectiveness of each treatment relative to the control. As previously defined, the characteristic (isolated) malignant cell lifetime, L = 1/K**_d_** = T/K*, is 63 days. For a malignant cell in a nodal environment the death rate is enhanced by the factor [1+N*] and by rituximab treatment. This is the factor E = [1+N*+K″g(c)] that multiplies K* (or K′) in Eq. 2, yielding a characteristic cell lifetime of L = T/K*E. The minimum value of E occurs at t = 0, so that E(0) = 1+N*(0)+K″g, yielding a ***maximum*** cell lifetime of L(0) = T/K*E(0). In the present experiments, N*(0) = 1, and g(c) = 1, yielding E(0) = K″ +2, and a maximum cell lifetime of L(0) = T/K*[K″ +2]. Using values for these parameters derived from ***only*** the early time (0 to 7 days) data, we obtain the results presented in Table 1.

**Table 1:** Cell lifetimes estimated from early time (t<7 days) data.

	Cell Death/Birth Rate	Cell Kill/Death Rate	Cell Lifetime (Days)
	K* or K′	K″ g	L(0)
Isolated cell	0.11	n/a	63
control	0.11	n/a	32
as- bcl-2	0.15	n/a	23
anti-CD-20	0.11	3.5	12
Combination	0.15	3.5	8

We note that the ratio of the “control” cell lifetime to as-bcl-2 lifetime is 1.4 and to anti-CD-20 is 2.7. The product of these ratios is 3.8, which is close to the ratio of control cell lifetime to combination drug cell lifetime (4.0), suggesting a multiplicative, synergistic effect of the combination treatment. However, the differences in estimated cell lifetimes are less pronounced when the later time data is included (see Figures 6b, c and d). While K* is the same, K′ = 0.09 and K″ = 2, with the result that L(0) for the four cases is 32, 39, 15 and 19 days, respectively. As observed earlier, there is no apparent long-term (>7 days) benefit to the initial administration of as-bcl-2 alone, but its early time influence delays re-growth of a tumor treated with rituximab. This behavior has been noted in previous experiments at Fox Chase Cancer Center [2] and elsewhere [6], [7], [8], although in the latter experiments repeated administration of as-bcl-2 yielded significantly greater efficacy. A single dose of each treatment was administered in the subject experiments to simulate the clinical regimen, but optimal dose scheduling is clearly an area for future experimentation and analysis.

This preliminary analysis is not inconsistent with both the experimental hypothesis and the basic assumptions in the parametric model: 1) average birth rates are relatively constant for each group of experiments; 2) the average malignant cell death rate increases and its lifetime decreases with the introduction of anti-CD-20 and in combination with as-bcl-2; and 3) the ratio of characteristic cell lifetime to that of the control, is comparable to the product of the ratios of independent therapies.

### Conclusions

An analysis of mouse tumor volume progression employing a parametric model has demonstrated an approach to the quantitative comparison of the efficacy of combination drug therapy relative to independent treatments. We have shown that this model may be used to combine efficacy data from independent treatments to predict the effectiveness of combination therapies.

At this point, there is insufficient data to compute statistical measures of the variation of K*, K′ and K″ for each animal in an experimental group, but comparison of the average tumor volume data with the model suggests that additional experiments would be worthwhile. Improved statistics would then provide greater confidence in the model predictions. In summary, we provide the following tentative answers to the questions posed at the outset:

1. A straightforward parametric model of tumor progression simulates the control experiments and the independent effects of as-bcl-2 and anti-CD-20 treatments.
2. The relative efficacy of each treatment compared to the control can be quantitatively derived with the model.
3. Synergistic effects of combined as-bcl-2 and anti-CD-20 treatment can be calculated without the need of additional parameters.
4. Key parameters derived from independent therapy experiments can be used to predict the effectiveness and optimization of combination therapies.
5. The relative effectiveness of as-bcl-2 and direct kill by anti-CD-20 can be quantified.

The effects of dosage and treatment frequency on tumor progression and regression is readily modeled with Eq. 2 using empirical data for g(c), the drug concentration factor. A review [9] of clinical studies comparing “maintenance” rituximab therapy with interventional treatment of NHL may also provide a useful clinical data set. For animals with intact immune systems, the interaction of cytotoxic T-cells or NK-cells with malignant B-cells, enabled by rituximab [10], can be modeled with an additional term in Eq. 2 and a separate (logistics-like) equation for the response of immune cell populations. Parametric models such as these should become useful in the planning of multi-parameter combination drug experiments and clinical trials [11], [12].

Parametric modeling of tumor response could be used in two different ways in clinical trials: 1) Interpretation of the results of multi-therapy trials in order to determine the particular effects of therapies individually and 2) Planning of future trials so that treatment dosages and timing are determined in a rational manner. If the model contains the primary biological mechanisms, it should be directly applicable to human data. However, the extrapolation of survival data in mouse experiments to the human case, particularly in subcutaneous xenografts, is problematic. In subcutaneous models, animals can accept extraordinarily large tumor burdens without ill effects (surpassing the 10% of body weight limit set by our institutional guidelines). Parametric modeling of tumor growth rates therefore provides a much more relevant assessment of treatment efficacy.

We also note that most tumors are under attack from the immune system and so all therapies act in combination with the immune system. As suggested, our method can indicate synergistic effects between treatments, and unravel the effect of cytotoxic treatments (anti-CD20) from those of pro-apoptotic treatments (anti-bcl2). Application of these methods to clinical data would not only elucidate the importance of each therapy and their potential synergies, but would form the basis for mathematical optimization that can be done prior to any treatment. Models may show, for example, that cytotoxic therapies are effective only at the early stages of treatment, and should be discontinued early, whereas anti-apoptotic treatments should be continuously administered Conversely, continuous anti-apoptic treatments could be shown to increase the effectiveness of later cytotoxic therapies, permitting lower dosages to be used. As pointed out in a recent publication [13], “mathematical modeling narrows down an infinite space of possible treatment strategies to a subset of strategies with the greatest potential that can then be validated in preclinical models before being introduced to patient care”. For example, if each disease state is characterized by only one parameter (K* in our model) with 10 possible values, and each of two treatments has 10 possible dose levels ranging from ineffective to toxic, there are a thousand possible experiments and associated outcomes in combination. If an immune response parameter is included, or three drugs are administered in combination, there are ten thousand possibilities to consider.

While modeling, in general, should have similar benefits for the understanding and treatment of other cancers, our model may be relevant to other blood cancers at best, and is designed specifically for the lymphomas.
